# Botanical Report

**Published:** 1811-11

**Authors:** 


### BOTANICAL REPORT.

Six Numbers of the Botanical Magazine have appeared since our last mention of this interesting work, which continues to be carried on with unabated spirit; and Mr. Edwards's drawings, if possible, continue to increase in merit.

Anacampseros *filamentosa* and *arachnoides* are separated from the genus Portulaca, by Dr. Sims, the propriety of which had been before suggested by Mr. Haworth. Neither of these appear to have been figured before, or even mentioned by any botanical author except Mr. Haworth.

Aristolochia tomentosa; a new species from the Hammersmith nursery, nearly allied to A. *sipho*; with which Dr. Sims seems to think it may have been confounded even by Michaux.

Erica *monadelphia*, a new species considered by Mr. Andrews as a variety of E. *banksii*, and omitted in the new edition of Hortus Kewensis. Very different from E. *monadelphia* of Andrews and Willdenow, which is E. *furfurosa* of Salisbury and of the Hortus Kewensis.

Rhapis *flabelliformis β*. A male plant. This is one of the humblest of the palms; all of which, from their being so seldom seen to flower with us, are subjects of curiosity.

Lachenalia *lucida* and *unicolor*; the latter appears to be badly named, three colours being distinguishable in the figure here given. The species of this genus appear to be very numerous; and the distinction of species from varieties perhaps not yet well understood.

Pothos *pentaphylla*, native of Guiana and St. Lucia, from Mr. Vere's collection, Kensington Gore.

Mesembryanthemum *minutum*; a species never described but by Haworth, and not taken up by Willdenow, though very distinct from *minimum*.

Rosa *bracteata*; a hardy ever-green Rose, brought from China by Lord Macartney, on his return from his celebrated embassy to the emperor. Though of so late introduction into Europe, this Rose has been twice before figured, by Wenland in his Hortus Herrenhausensis, and by Ventenat, from the garden of M. Cels. Communicated by Messrs. Malcolms, nurserymen, at Kensington.

Idia *monadelphia β*, and *fucata*.

Drimia *lanceæfolia:* considered as a species of Lachenalia by all botanists before Mr. Ker. This number contains an enumeration of all the species of this genus known to Mr. Ker.

Allium *bisulcum* of Ridouté; the only author who has before noticed this species.

Neottia *speciosa.* Native of the West Indies; an ornamental plant in our stoves, flowering in the midst of winter.

Eriospermum *latifolium* ($\alpha$). With an enumeration of the known tspecies of this very curious genus. From Mr. Knight's nursery, in he King's Road.

Crocus *sulphereus* $\beta$. One of the least splendid of the species of this harbinger of the spring.

Allium *ampeloprasum;* native of the Levant, of Portugal, and of Home's Island in the Bristol Channel. Mr. Ker suspects it to be the origin of the common cultivated Leek.

Geranium *ibericum;* a species known to Tournefort, but only lately n oduced into Europe. Communicated by Messrs. Whitley and Brame, nurserymen, at Old Brompton and at Fulham. It makes a remarkably beautiful drawing.

Cytisus *divaricatus* ($\beta$), a smooth-leaved variety, and rather an ornamental shrub, requiring to be protected from severe frost. From Messrs. Loddige.

Tussilago *fragrans;* a late introduction among us, though commonly cultivated in France for the sake of its odoriferous flowers. From the Hammersmith nursery.

Podalyria *cupinoides;* a very rare species, and still more rarely seen in flower. Mr. Loddige, who has been in possession of the plant several years, never was so fortunate as to have it in flower before.

Tulipa *clusiana.* This article contains an enumeration of the species, as known to Mr. Ker.

Carex *fraseriana.* A new and very remarkable species, with leaves resembling a liliaceous plant. Found in North Carolina by the late Mr. Fraser.

Trichonema *caulescens.* From the Hammersmith nursery.

Iris *ruthenica* $\beta$. This plant was supposed to be Iris *verna;* but, from the account here given by Mr. Ker, it seems probable that I. *vernæ* of Gronovius, Linnæus and Miller, is only a variety of *cristata;* or, at least, it is much nearer akin to that species than to the one here figured; which has usually passed in our nurseries for it.

Pultenæa *daphnoides.* One of the first, and now one of the most common, of the plants from New South Wales.

Zieria *smithii.* Native of New South Wales. A shrub named by Dr. Smith, in honour of Mr. Zier, a learned botanist, of German extraction, but who lived and died in this country.

Pittosporum *tobira.* A fine evergreen tree, with sweet-scented flowers; native of China; from the collection of Messrs. Malcolm and Sweet, at Stockwell common.

Stapelia *reclinata;* from the collection of Messrs. Walker, at Stockwell. We have no doubt but that this genus, now so extensive, will be hereafter divided into several.

Bignonia *grandiflora.* This plant is really a great acquisition to our gardens.

gardens. It is not improbable but that it may be found as hardy as B. *radicans;* but if not, it promises to be a splendid ornament of our green-houses. The drawing of this plant is very characteristic, and one of the most beautiful in the work.

Erica *odorata.* The heaths have few of them any scent, but this and *fragrans* make two exceptions, both of them having a powerful and agreeable perfume; that of the former is compared by Dr. Sims to a mixture of roses and honeysuckles. It is likewise very elegant in its growth.

Ruellia *formosa.* A highly ornamental stove plant, producing splendid scarlet flowers most part of the summer. From Messrs. Whitley, Brame, and Martin's.

Lachenalia *contaminata.* L. *lucida, unicolor,* and *contaminata* appear to be nearly allied, in their flowers, though their foliage is sufficiently different.

Uvularia *sessifolia.* Native of North America, introduced by Messrs. Fraser and Sons.

Smilacina *borealis.* This is the same plant as was figured in the former edition of the Hortus Kewensis, under the name of Dracœna borealis. The one before published under this name as a supposed variety, Mr. Ker is convinced, upon having seen both, is a different species, which he calls *umbellata.* Dianella ensifolia *α.* An old stove plant; but its native country and time of introduction, both uncertain.

